# Psychological distress related to the emerging COVID-19 pandemic and coping strategies among general population in Egypt

**DOI:** 10.1186/s42506-021-00100-2

**Published:** 2022-01-12

**Authors:** Manal Mohamed Elkayal, Mahmoud Abdel Hameed Shahin, Rasha Mohammed Hussien

**Affiliations:** 1 Department of Psychiatric and Mental Health Nursing, Faculty of Nursing, Zagazig University, Zagazig, Egypt; 2 Department of Critical Care Nursing, Mohammed Al-Mana College for Medical Sciences, Dammam, Saudi Arabia; 3 Department of Psychiatric and Mental Health and Community Health Nursing, College of Nursing, Qasim University, Buraydah, Saudi Arabia

**Keywords:** Coping, Coronavirus, COVID-19, Pandemic, Psychological distress

## Abstract

**Background:**

Psychological distress is considered a threat to the mental health of human beings. This research was conducted at the beginning of the emerging COVID-19 pandemic, when most people had limited knowledge about coronavirus, mode of transmission, associated manifestations, with uncertainty about treatment, vaccine, future life, and coping capacity. This study examined the nature of the psychological distress related to the emergence of the coronavirus disease 2019 (COVID-19) pandemic and coping strategies adopted among the general population in Egypt.

**Methods:**

This was a descriptive, cross-sectional study assessing a convenience sample consisting of 312 participants from the general population in Egypt. Data were gathered as online responses to a questionnaire which incorporated a sociodemographic datasheet, psychological distress scale, and ways of coping scale.

**Results:**

Forty-two percent of the participants showed severe psychological distress and 26% showed mild to moderate psychological distress. There was a strong positive correlation between the distress score and the overall coping score—that is, the higher the distress, the more ways of coping were adopted (*p* < 0.001). This study also showed that the methods of adaptation used by most of the population were based on emotional coping strategy. The most adaptive people were those who work in the health field and the residents in the cities with a monthly income sufficient enough to meet their needs; better adaptation methods were also seen among both divorced and highly educated people. We also found a significant relationship between sociodemographic characteristics except for sex and overall coping methods (*p* < 0.001). Further, significant relationships between sociodemographic characteristics and psychological distress were observed (*p* < 0.001).

**Conclusion:**

Most of the study population as a sample of the general population in Egypt reported suffering from varying degrees of psychological distress during the COVID-19 crisis. However, the more severe an individual’s level of psychological distress, the greater their adaptation ability was. This study focuses light on the importance to provide appropriate interventions against COVID-19-related stresses and equipping people with suitable strategies for coping with the COVID-19 pandemic.

## Introduction

A novel viral disease that emerged first in China in late 2019 and subsequently was labeled as coronavirus disease 2019 (COVID-19) by the World Health Organization (WHO) in February 2020 has spread to affect people all over the world, eventually meeting the organization’s criteria to be designated as a pandemic. A significant increase in global mortality and morbidity rates as a result of this pandemic has occurred [1]. Similarly, the level of stress among the public has continually grown despite efforts made by both the WHO and local public health authorities to contain the COVID-19 outbreak and its consequences [2]. In response, the WHO Department of Mental Health and Substance Use created and refined a list of considerations that could be used to support mental and psychosocial well-being in different target groups during the outbreak period of a communicable disease, including decreasing the time spent reading, listening, or looking at information about the condition, which increases levels of distress and anxiety [2]. The limitless dissemination of information and news about an outbreak can lead nearly everyone to experience heightened anxiety levels. Therefore, it is recommended that individuals seek information only from trusted sources once or twice daily as this is believed to be sufficient for most people to identify and apply practical steps to protect both themselves and their loved ones [2].

Coronavirus has caused negative effects on mental health among people in all places as a result of the precautionary measures enacted and the fear of easy transmission of the SARS-CoV-2 virus between people, especially given that it is purported to be transmitted through droplets expelled during talking, coughing, and sneezing and possibly via air currents as well. Psychological distress characterized mainly by depression and anxiety symptoms is a common mental health problem in the community [3]. Different levels and types of psychological distress can be experienced by members of the general population, including anxiety, nervousness, depression, inattention, sleep problems, and fear of injury or infection. The onset of psychological disorders has also been reported to have occurred during similar outbreaks [4]. These symptoms often occur simultaneously with common somatic complaints [5].

The isolation and lockdown states have been prolonged to varying degrees as a result of reaching new peaks in mortality and morbidity rates every day. Therefore, recreational opportunities for people have been lessened and the financial crisis has been building, with a forthcoming exponential increase in mental health issues anticipated. This has led to varying levels of psychological distress in many people. The lockdown strategy adopted by most countries in response to the COVID-19 pandemic led to negative impacts on mental health including insecurity about the future and work loss-related anxiety. Moreover, it has had a potential exacerbating impact on existing chronic diseases and affected persons may show psychiatric symptoms possibly related to the interaction between mental disorders and disease-related immunity [6].

During the COVID-19 pandemic, the practice of remaining physically apart or “social distancing” has been suggested as an imperative to ensuring health and survival. Of course, when humans are quarantined and/or kept in one place, they lose privileges that they once took for granted and had the full freedom to do at socially acceptable times, such as eating at restaurants; attending social gatherings, parties, or protests; going out with friends; working at their job of choice; and going to the gym [7].

Consequently, as it is the nature of humans to adapt to events ongoing in their lives, people resorted to ways of coping with the restrictions put in place to mitigate the pandemic. Such coping strategies include both behavioral and psychological efforts aimed at mastering, tolerating, or minimizing stressful events [8]. Positive coping has been associated with reductions in stress levels and thus results in greater well-being, while negative coping is often related to stress enhancement, resulting in increased psychological distress [9]. The extent to which coping increases or reduces psychological distress is highly dependent upon the collection of stressors, adaptation manners, and resources and the coping responses chosen to combat such stressors [10]. Evidence has suggested that the lifestyle quality of the general population is influenced by their coping styles against stress [11].

Muhonen and Torkelson [12] suggested that the use of problem-focused coping tends to be more beneficial to ensuring the health of an individual than that of emotion-focused coping; this is because the core aspect of problem-focused coping involves changing the way people view the source of their stress and developing ways for how to solve it, while emotion-focused coping revolves around avoiding stressors for a while but ultimately not eliminating the effects of the stress.

Even quarantined or isolated individuals, with connections to a small circle of social contacts, can continue to interact during everyday duties and perform self-care despite their dire circumstances. These individuals can, for example, participate in a range of activities along with hobbies and mentally difficult tasks such as solving mental puzzles, reading, listening to music, singing, enjoying an instrument, watching pleasant programs on television, gaining knowledge of a language, taking part in web games, and planning for a better quality of life following the pandemic. Finding approaches by which to safely interact with others and respect their altered lifestyles during mass trauma is a strong predictor of improved psychological well-being [13]. To date, given its novelty, this problem has not been widely addressed by researchers, especially those from Egypt. The current study aimed to investigate the profiles of psychological distress related to the COVID-19 pandemic among the general population in Egypt and determine the coping strategies being used.

## Methods

### Research design

This study employed a descriptive cross-sectional research design.

### Setting and procedures

The study was carried out on a sample of the general population in Egypt assessed during the COVID-19 pandemic outbreak. Like other countries in the world, the Egyptian government recommended the public minimize the conduct of face-to-face interactions and gatherings for any reason. Using the researchers’ relations and social networks, we sent out an electronic consent form with a questionnaire inviting individuals to participate in the study. The link was disseminated to the sample population through smart applications and social media. The data collection questionnaire required approximately 30 to 45 min to complete and the data collection process took place over 2 months from 1 April 2020 to 1 June 2020.

### Sampling

A convenient sample of 312 people from the general population in Egypt was recruited. Egyptians who were 18 years old and willing to participate in the study were eligible for inclusion in the current study. The sample size was calculated according to the population of Egypt, at a 90% level of confidence, with 5% confidence limits, a 50% anticipated frequency, and a design effect value of 1.0. Using the open-source OpenEpi version 3.01 software program [14]. The required sample size was determined to be 271 subjects; however, the sample was increased by 15% to 312 subjects to better ensure the achievement of the targeted confidence level.

### Data collection tools

We deployed a self-administered questionnaire form consisting of the following three parts:

*A sociodemographic datasheet* was designed by the researchers to assess the sociodemographic characteristics of the study sample. It included questions on age, gender, level of education, marital status, occupation and the nature of the work performed, whether the respondent was working in the health care sector, monthly income, and the number of family members.

*The Kessler Psychological Distress Scale (K10).* The original version of the K10 by Kessler et al. [15] was translated to Arabic then back-translated. K10 represents a 10-item scale that assesses the frequency of nonspecific psychological distress symptoms experienced during the last 30 days. This self-report measure primarily includes questions about anxiety and despair or depression symptoms. Participants are asked to answer on a 5-point scale (1 = none of the time to 5 = all of the time) and the total score can range from 10 to 50 points, with higher scores suggesting a greater degree of distress. Meanwhile, the following cutoff scores were chosen by Andrews and Slade [16] to estimate the psychological misery and distress level as follows: 10 to 15 points suggests low distress; 16 to 21 points suggests moderate distress; 22 to 29 points suggests high distress, and 30 to 50 points suggests very high distress. According to this approach, respondents with a total score greater than 22 points have a high risk of psychiatric distress or mental instability [16].

*Ways of Coping Scale (WCS)* [17]. This section was theoretically based on the revised, German-language version, aiming to draw attention to the question of how people are coping with COVID-19 from a psychological perspective. The German model is distinguished by its differentiation between problem-focused and emotion-focused coping. Besides, concrete behaviors are part of the survey, with a particular focus on tangible instances of behaviors like washing and disinfecting the hands. In the questionnaire, 28 questions concerning problem-focused and emotion-focused strategies for coping with the COVID-19 crisis were included alongside 7 items about concrete behaviors performed or adopted. Additionally, 4 questions focusing on the respondent’s trust of politicians, authorities from the medical field, and medical companies are included.

Separately, we added some items to the WCS to secure other data pertaining to problem-focused and emotion-focused coping strategies adopted during the COVID-19 pandemic. These include item number 13 “I try to maintain social distancing” (problem-focused) and items 5, 12, and 15 through 18 “I've been making jokes about it”; “I've been doing something to think about it less, such as going to movies, watching TV, reading, daydreaming, sleeping, or shopping”; I've been looking for something good in what is happening”; “I've been accepting the reality of the fact that it has happened”; “I've been trying to find comfort in my religion or spiritual beliefs”; and “I've been expressing my negative feelings” (emotion-focused). The WCS relies on a 5-point Likert scale and was translated into the Arabic language using the translation–back-translation method to ensure its validity, which was tested by a committee of five members in the field of psychiatric and mental health nursing. The scores of the scale items were scored as 0, 1, 2, 3, and 4 points, respectively, for the responses never, rarely, sometimes, usually, and always; then, the scores of items were summed up and the total was divided by the number of items, yielding a mean score for each subscale.

For categorization, scores were transformed into percentages. The respondent was considered to have a high coping ability if the percentage score was 60% or greater (corresponding to usually/always) and low coping ability if the percentage score was less than 60% (corresponding to sometimes/rarely/never). For every subject, the coping type or manner marked by the greatest percentage score was viewed as the type that most predominantly used by that respondent.

#### Validity and reliability

The translated final tool was reviewed by a panel of five experts in the field of psychiatric and mental health nursing to test the content and face validity of the questionnaire, which was deemed acceptable. Reliability of the final questionnaire format was determined using the Cronbach’s alpha coefficient test for the K10 (Cronbach’s α = 0.94), the problem-focused WCS subscale (Cronbach’s α = 0.96), and the emotion-focused WCS subscale (Cronbach’s α = 0.74).

Similarly, a test-retest analysis was conducted and reflected good reliability of the scales the K10 (0.78), the problem-focused WCS subscale (0.81), and the emotion-focused WCS subscale (0.75). At the same time, confirmatory factor analysis of the WCS was carried out to assure that the model fits. The findings revealed that the model fits as reflected by the *P* value using JASP software (*P* > 0.05). The factor model test of the chi-square reflected (*X*^2^ = 66.75, df = 11, and *P* value of 0.076).

#### Pilot study

A pilot study was carried out before conducting the main investigation to test the clarity of the scales and the feasibility of this study. The pilot study was conducted involving 27 persons who represented 10% of the calculated required sample size. From the pilot study results, it was found that the average time required to fill in the tool with its three scales ranged from 30 to 45 min according to the respondent’s level of understanding and cooperation. Based on the pilot results, the study tools were finalized and the pilot subjects were thereafter excluded from the main study sample. Participants in the pilot study were informed prior to inclusion that they would not be allowed to participate in the main study.

### Ethical considerations

Before initiating the study and data collection processes, the research proposal was approved by the ethical committee of the Faculty of Nursing, Zagazig University. The information of the study participants was kept confidential throughout the study by leaving them anonymous and allocating codes to the questionnaires. Given this practice, it was requested that participants provide honest answers. An explanation about the study encompassing all necessary data about the study objectives, procedures, benefits, and potential risks was offered clearly to participants ahead of beginning the questionnaire and approval for study inclusion (informed consent) was obtained from each participant before their involvement. Participants were asked to answer a polar (yes–no) question to confirm their willingness to participate in this study voluntarily. After confirmation of their willingness and interest, the participant was directed to complete the self-report questionnaire. All ethical principles regarding medical research involving human subjects, in accordance with the Declaration of Helsinki, were followed [18]. Official permission to use, modify, and translate the scales were granted by the original authors.

### Statistical analysis

Descriptive statistics for categorical variables, such as those relating to sociodemographic variables, were presented using numbers and percentages, while, the distress score and different coping scores were presented using means and standard deviations. Correlations between coping scores and numeric variables were performed using Pearson’s correlation, while those involving ordinal variables (age and education) were conducted using Spearman’s correlation.

Meanwhile, the comparison of coping scores among different categories was completed using the independent *t* test and one-way analysis of variance. A multiple linear regression model was adopted to study the association between the K10 score and the WCS mean scores. Statistical significance was set at *p* < 0.05. The analysis was performed using the Statistical Package for the Social Sciences version 25 software program (IBM Corporation, Armonk, NY, USA) [19]. No missing data were expected as each question required an answer before the respondent was able to move on to the next question.

## Results

A total of 312 people were included as participants in this cross-sectional study. Table 1 presents the sociodemographic data of the study subjects of the total study population, 57.4% were males and 42.6% were females, while subject ages ranged between 18 and 67 years with most of them aged 18 to 37 years (52.6%). The highest percentage of the participants had obtained a university education (46.5%), while 4.8% were not educated but can only read Arabic or that someone has read the questionnaire to him. The majority of study subjects were employed (70.5%), including 42.7% who were working in public sectors and 45% who were working in the private sectors. Most participants did not work in the health care field (89.1%). Around two-thirds of the participants lived in urban areas and had a sufficient monthly income (61.5% and 60.6%, respectively).

**Table 1:** Sociodemographic characteristics of the study sample of the general population during COVID-19, Egypt 2020 (*N* = 312)

Sociodemographic data	Frequency	Percentage
**Sex**		
Male	179	57.4
Female	133	42.6
**Age**		
18–27 years	81	26
28–37 years	83	26.6
38–47 years	69	22.1
48–57 years	42	13.5
58–67 years	37	11.9
**Education**		
Not educated	15	4.8
Able to read and write	31	9.9
Intermediate education	101	32.4
University degree	145	46.5
Postgraduate studies	20	6.4
**Marital status**		
Single	66	21.2
Married	224	71.8
Divorced	7	2.2
Widow	15	4.8
**Employment**		
Employed	220	70.5
Not employed	92	29.5
**Nature of work (among those who are working)**		
Governmental sector	94	42.7
Private sector	99	45
Daily worker	27	12.3
**Working in the health care field**		
No	278	89.1
Yes	34	10.9
**Residence**		
Urban	192	61.5
Rural	120	38.5
**Number of family members**		
2–4	141	45.2
5–7	151	48.4
≥ 8	20	6.4
**Monthly income**		
Sufficient	189	60.6
Insufficient	123	39.4

Table 2 reports the mean scores of psychological distress and ways of coping scales recorded by participants during the last 30 days. The overall psychological distress mean score was 26.51 ± 9.433 points. The total emotion-focused WCS subscale score was higher (66.86 ± 7.568) than the problem-focused WCS subscale score (50.65 ± 13.011). Meanwhile, the overall WCS score was 117.51 ± 14.7301 points.

**Table 2:** Psychological distress and Ways of Coping Scale scores for the study sample of general population during COVID-19, Egypt 2020 (*N* = 312)

Scale		Mean	SD
Kessler Psychological Distress Scale	Total mean score^a^	26.51	9.433
	Average score	2.651	0.9433
Problem-focused ways of coping	Total mean score^b^	50.65	13.011
Emotion-focused ways of coping	Total mean score^b^	66.86	7.568
Ways of Coping Scale (WCS)	Total mean score^b^	117.51	14.7301

*SD* standard deviation

^a^Five-point Likert scale (1 = none of the time; 5 = all of the time)

^b^Five-point Likert scale (1 = strongly disagree; 5 = strongly agree)

Table 3 shows the association between sociodemographic data and psychological distress scores. There was a statistically significant relationship between some sociodemographic characteristics and psychological distress. The mean psychological distress score was significantly higher in correlation with female sex, employed status, working in the health care sector, living in an urban area, and having insufficient monthly income (*p* ≤ 0.001). Moreover, there was a weak negative correlation between total psychological distress and age (correlation coefficient = − 0.156).

**Table 3:** Association between sociodemographic factors and psychological distress scores during COVID-19, Egypt, 2020

Sociodemographic data	***n***	Mean	SD	***p*** value
**Sex**				**< 0.001^a^
Male	179	24.9	8.8	
Female	133	28.6	9.8	
**Employment**				**< 0.001^a^
Employed	220	27.9	9.2	
Not employed	92	23.3	9.3	
**Work field**				0.221 ^b^
Governmental sector	94	28.7	10.2	
Private sector	99	27.8	8.2	
Daily worker	27	25.2	8.3	
**Working in the health care sector**				**< 0.001^a^
Yes	34	36.3	7.4	
No	278	25.3	9	
**Residence location**				**0.001^a^
Urban	192	27.9	8.8	
Rural	120	24.3	9.9	
**Monthly income**				**< 0.001^a^
Sufficient	189	25.1	9	
Insufficient	123	28.7	9.7	
**Marital status**				0.305 ^b^
Single	66	26.1	8	
Married	224	26.5	9.6	
Divorced	7	33	4	
Widow	15	25.4	13.2	
**Number of family members**				0.120 ^b^
2–4	141	28.2	8.7	
5–7	151	25.2	9.7	
≥ 8	20	24.4	10.2	
**Age (Spearman’s correlation coefficient)** = − 0.156				**0.006 ^c^
**Educational level (Spearman’s correlation coefficient)** = 0.055				0.330 ^c^

^**^ Significant at the 0.01 level (two-tailed)

*n* number, *SD* standard deviation

^a^Independent *t* test was used

^**^ Significant at the 0.01 level (two-tailed)

^b^One-way analysis of variance was used

^c^Spearman's correlation was used

Table 4 shows the association between the ways of coping and sociodemographic factors. Higher mean scores on the problem-focused WCS subscale were significantly associated with employed, working in the governmental sector, working in the health care sectors, living in an urban area, had a sufficient monthly income, being divorced, and having two to four family members. On the other hand, higher mean scores on the emotion-focused WCS subscale had a statistically significant association with employment in private sectors and being single or divorced (*p* ≤ 0.001). A significant negative correlation between age and emotional-focused WCS score was detected, while a significant positive correlation was noticed between the educational level and the problem-focused WCS subscale score.

**Table 4:** Relationship between sociodemographic characteristics and mean Ways of Coping Scale (WSC) subscale scores during COVID-19 in Egypt

Sociodemographic data	Problem-focused coping			Emotional coping		
	Mean	SD	*p* value	Mean	SD	*p* value
**Sex**			0.189 ^a^			0.284 ^a^
Male	3.6	0.8		3.4	0.4	
Female	3.8	1		3.4	0.4	
**Employment**			**< 0.001^a^			0.059 ^a^
Employed	3.8	0.8		3.3	0.4	
Not employed	3.3	0.9		3.4	0.4	
**Work field**			**< 0.001^b^			**< 0.001 ^b^
Governmental sector	4.2	0.6		3.3	0.4	
Private sector	3.8	0.7		3.5	0.4	
Daily worker	2.6	0.7		3.2	0.3	
**Working in the healthcare sector**			**< 0.001^a^			0.054 ^a^
Yes	4.6	0.4		3.2	0.4	
No	3.6	0.9		3.4	0.4	
**Residence**			**< 0.001^a^			0.374 ^a^
Urban	4.1	0.5		3.4	0.4	
Rural	3	0.8		3.3	0.4	
**Monthly income**			**< 0.001^a^			0.182 ^a^
Sufficient	4	0.6		3.4	0.4	
Insufficient	3.2	1		3.3	0.4	
**Marital status**			**< 0.001^b^			**< 0.001 ^b^
Single	3.5	0.8		3.6	0.4	
Married	3.8	0.8		3.3	0.4	
Divorced	4.4	0.8		3.6	0.3	
Widow	2.9	1.1		3.3	0.4	
**Number of family members**			**< 0.001^b^			0.691 ^b^
2–4	4	0.8		3.4	0.4	
5–7	3.5	0.8		3.4	0.4	
≥ 8	2.6	0.9		3.4	0.4	
**Age (Spearman’s correlation coefficient)**	− 0.105		0.064 ^c^	− 0.198		**< 0.001 ^c^
**Educational level (Spearman’s correlation coefficient)**	0.52		**< 0.001^c^	0.088		0.120 ^c^

^a^Independent t-test was used

^**^Significant at the 0.01 level (two-tailed)

^b^One-way analysis of variance was used

^**^Significant at the 0.01 level (two-tailed)

^c^Spearman’s correlation was used

^**^Significant at the 0.01 level (two-tailed)

*SD* standard deviation

The results in Table 5 reveal the existence of a highly significant relation between all categories of psychological distress and problem- and emotional-focused coping. An increase in psychological distress was significantly associated with an increase in problem- and emotional-focused WCS subscales mean scores (*p* < 0.01); however, the values were higher for the emotional-focused WCS mean scores.

**Table 5:** Association between psychological distress and mean Ways of Coping Scale (WCS) subscale scores during COVID-19 in Egypt

Psychological distress categories	Problem-focused coping		Emotional coping	
	Mean	SD	Mean	SD
**< 20 points** (likely to be well)	49.61	11.27	61.28	7.27
**20–24 points** (likely to have mild psychological distress)	55.62	12.6	66.59	5.95
**25–29** points (likely to have moderate Psychological distress)	55.88	12.9	62.23	8.27
**≥ 30 points** (likely to have severe psychological distress)	59.29	13.11	66.1	7.17
*F* value	11.45		10.89	
*p* value	< 0.01^*^		< 0.01^*^	

^*^Significant at the 0.05 level (two-tailed)

*SD* standard deviation

Table 6 illustrates a prediction equation for WCS subscale mean scores that was developed from the linear regression analysis involving the mean scores of psychological distress and the WCS subscales. The mean score of psychological distress was a significant positive predictor of the WCS subscale scores; however, an increase in psychological distress was associated with adopting more emotion-focused ways of coping (*r* = 0.59) than adopting problem-focused ways of coping (*r* = 0.123). The following multiple linear prediction equations were formulated to predict the psychological distress mean score:

$$ y1=1.722+0.198x $$

$$ y2=1.667+0.117x $$

*y*1 is the emotion-focused WCS subscale mean score, *y*2 is the problem-focused WCS subscale mean score, and *x* is the psychological distress mean score.

**Table 6:** Best-fitting multiple linear regression model for WCS scores using psychological distress among the study sample of general population during COVID-19, Egypt, 2020

Model	Unstandardized coefficients				***R***^**2**^	***F***	Sig.
	*B*	Std. error	*t*	Sig.			
Emotion-focused ways of coping	1.722	0.089	19.404	0.000	0.59	19.322	*0.000
	0.198	0.030	6.588	0.000			
Problem-focused ways of coping	1.667	0.079	21.155	0.000	0.123	43.407	*0.000
	0.117	0.027	4.396	0.000			

Independent variable: psychological distress mean score

^*^Correlation is significant at the 0.01 level (two-tailed)

## Discussion

The stress caused by the COVID-19 epidemic has had a critical impact on individuals and whole social groups alike. Different levels of psychological crisis can be experienced by individuals. This study aimed to investigate the severity of psychological distress and the coping strategies adopted among a sample of the Egyptian population during the COVID-19 pandemic.

The results of this study revealed that more than two-fifths of participants were experiencing severe psychological distress, with higher mean scores for feeling that everything was an effort, feeling nervous, and feeling restless or fidgety reported, while lower mean scores were recorded for feeling so nervous that nothing could calm the respondent down and feeling worthless, respectively. These results could be attributable to the drastic social distancing measures introduced by the government as a result of the fear of community spread of the SARS-CoV-2 virus. These measures included banning activities that involve crowds of people, closing schools and universities, sheltering in place, canceling major concerts and sporting events, closing local businesses, and suspending civil and religious ceremonies including funerals and weddings. These measures were aimed at minimizing the spread of the SARS-CoV-2 virus and authorities and stakeholders appealed to the public to avoid gatherings and meetings as much as possible [1].

However, these measures increase the risks and potential severity of loneliness, psychological distress, isolation, and anxiety as supported by Xiang et al. [4] who reported that the daily life of general populations is affected negatively by such an event. Different elements of psychological distress including depression, fear of infection, nervousness, anxiety, inattention, and sleep problems have been experienced by the general population as an epidemic continues. On the same line, Galea et al. [20] reported that the COVID-19 pandemic has forced people to social distancing and isolation; encountering health and economic crises, irrespective of profession, origin, and religion. Patients, health professionals, and the general public are under unprecedented mental pressure that may result in a spectrum of short- and long-term psychological health issues like anxiety, stress, depression, panic attack, and post-traumatic stress disorder.

Wang et al. [21] concluded that, at the time of similar epidemics or catastrophic events such as diseases and natural disasters, some psychological issues usually occur among the public and may persist for a long period. Other evidence contends that the strong infectious nature of the SARS-CoV-2 virus, lack of specific medications or vaccines, and the increased mortality rate among severe cases have led to a significant threat to human health and life [22]. Furthermore, COVID-19 has had a marked effect on the mental health of the global population, resulting in different degrees of emotional problems. In the same vein, a recent study has reported rates of psychological stress resulting from quarantine as a preventive measure against pandemics ranging from 24.6 to 56.0% [23]. Some research from Spain has also suggested a correlation between the implementation of social distancing and preventive measures and an increased level of anxiety [24].

The results of the current study indicated that the total psychological distress was higher in correlation with female sex, employed status, working in the health care sector, living in an urban area, and having an insufficient monthly income. These findings could be explained by the high level of awareness of people with higher education, which results in more distress. On the same line, Shrestha et al. [25] reported in their study in Nepal that female participants and health professionals had a higher risk of developing psychological distress during the COVID-19 pandemic. They also found that the education level and the income level had an inverse correlation with the level of psychological stress of the Nepalese community. On the contrary, their study was inconsistent with our findings regarding the elevated psychological distress level in rural areas. Likewise, healthcare personnel may experience psychological distress due to close contact with patients with COVID-19 for long hours, fear of transmitting the infection to their families, knowing someone who has died from the disease, in addition to the attached stigma which increases their feeling of psychological distress, fear, and anxiety. Accordingly, Wu et al. [26], many factors have influenced the psychological impact of the pandemic among health care workers, including the lack of proven therapies or a vaccine, shortages of health care resources, and uncertainty about the duration of the crisis. Social distancing has also a diverse effect on health care workers, resulting in distress due to the desire to be close to their families and worries of possible illness.

There was a weak negative correlation between participants’ level of distress and their age; psychological distress was higher among those who were younger. This result can be interpreted by the habits of young adults to obtain too much information from social media and from reading previous research. These results were supported by Zhang et al. [27] from Brazil, who reported that individuals who were females, younger, more educated, or exercised less had more psychological distress. In congruence with the current study that found higher psychological distress among employed individuals, who may lose their work due to the lockdown, and people having an insufficient monthly income, subsequently may suffer more financial difficulties due to the pandemic, Brooks et al. [28] found that poor mental health also resulted as a consequence of lockdown, which led to financial pressure due to economic fallout. Concerning marital status from a Saudi cross-sectional study, Elhessewi et al. [29] reported that psychological distress was significantly higher among younger age groups and unmarried individuals. These results may be attributed to the fact that young individuals, as well as unmarried people, may suffer a higher level of psychological distress in the presence of a pandemic disease or a disaster that may threaten their life compared to others, as they may feel that they are still in the prime of life, they have more hope and attachment in life, and they have not accomplished What is supposed to be accomplished; Therefore, they had felt more anxious and stressed than others as a result of the emerging COVID-19 pandemic and its health consequences on them. In addition, several explanations have been offered for the level of distress among young people including access to a huge amount of information via social media and the stronger impact of the ban and lockdown on young people.

Sex had also had a significant effect as another similar study has reported a higher risk of psychological distress has existed during the COVID-19 pandemic in women [30]. Females usually feel more anxious about their health and the health of their children. On the contrary, however, Mihashi et al. [31] concluded that the risk of psychological distress was higher in men.

The current results revealed that the mean scores psychological distress were higher in participants with a family composed of two to four members as compared with those with families of other larger sizes. This might be because the participants might be younger and have children they are concerned about or fearful about their health. This finding coincides with those reported by Taylor et al. [32], who suggested that a greater risk of psychological distress exists for people with children during pandemics. On the contrary, Naushad et al. [33] reported no association between the psychological impact of the pandemic and having children.

Our results also suggest that participants in urban residences have higher mean scores of psychological distress than those in rural areas. The situation of epidemics in urban cities is usually more serious due to the increased density of people and crowdedness; moreover, residents of urban areas are more educated. However, rural participants pay more attention to the sickness of relatives and neighbors and show more sadness and empathy toward patients, as conveyed in another study [34].

Coping is defined as an array of actions and thoughts adopted by an individual to deal with a stressful occasion or event. Our study found that emotion-focused coping was more widely used by the participants than problem-focused coping. As a result of the impact of the COVID-19 on people’s emotions, coping strategies are also likely to evolve over time. Our study was conducted from April 2020 to the beginning of June 2020; during this period, the population increasingly became aware of the presence and severity of the COVID-19 crisis as more information continued to emerge and SARS-CoV-2 infections spread. It is frequently crucial and considered highly effective to confront the stressor directly, instead of handling the feelings evoked from the stressor.

The current study results showed that the study population had largely already adopted protective measures such as the frequent use of sanitizers, hand-washing, wearing masks, and maintaining physical distance, which indicates an increased level of concern and desire to avoid the spread of the SARS-CoV-2, likely as a result of the increasing mortality and morbidity rates reported each day. This result was congruent with those findings of Folkman and Lazarus [35], who similarly described the use of problem-focused coping and emotion-focused coping as two strategies used to deal with distress.

Likewise, Goodwin et al. [36] explained conservation values such as tradition, conformity, and security as factors that affect public worry. People who subscribe to conservation values would apply preventive measures into practice, whereas individuals with contrasting values (e.g., great self-direction, stimulation, and hedonism based totally on Schwartz’s model) pay less attention to the greater good and focus on perpetuating behaviors they find favorable.

The emotion-focused items draw attention to the timespan that is perceived to relate to the COVID-19 pandemic. The study participants tended to have hope for a miracle and have been trying to find comfort in religion or spiritual beliefs. Alwala [37] reported a strong association between religious activities or spirituality and good health, more recoveries, and lower mortality rates; Furthermore, transcendent experiences and beliefs, such as spirituality morals and values, significantly facilitate resilience. Just by imparting a direct avenue through which adversities are recast into meaningful narratives, achieving a look beyond oneself and one’s continuous plight, people can gain the chance to develop hope and optimism for the future.

The present study indicated that emotion-focused ways of coping were more widely used by the participants than problem-focused coping. This result was inconsistent with those of Gerhold [38] who reported that problem-focused coping strategies were rated higher than emotion-focused strategies with respect to their use among 1242 respondents experiencing the COVID-19 pandemic in Germany. This discrepancy may be due to a difference in the culture between Egypt and Germany; also, at the time of the study by Gerhold, the confirmed COVID-19 cases and deaths in Germany were greater than those in Egypt [39], which may have led to an increase in the risk perception of the German population regarding COVID-19 and, thus, greater use of problem-focused coping.

In general, the results of the current study revealed a highly significant relation between all categories of psychological distress and problem-and emotion-focused coping. In addition, psychological distress was a statistically significant positive predictor of WCS subscale scores. Considering the increase in psychological distress, higher psychological distress was associated with greater use of emotion-focused rather than problem-focused coping strategies. This was supported by a previous study that concluded that the general population usually responds differently, either positively or negatively, when faced with stress or traumatic experiences [11]. The degree of emotional impact varies among populations when infectious diseases appear. People can also easily adopt maladaptive techniques to maintain posttraumatic stress disorder symptoms such as invasion, arousal symptoms, and robust devastating emotions.

In general, the current study revealed a significant negative correlation between the age of participants and the emotional-focused WCS score, while a significant positive correlation was noticed between the educational level and the problem-focused WCS subscale score. Our findings are similar to those of Bish and Michie [40], who reported an association between the chance of adopting protective behaviors and being younger and more educated. Also, Gerhold [38] revealed that older respondents show a reduced chance of using emotion-focused coping techniques. However, no significant correlations were determined between the use of problem-focused techniques and age.

### Limitations

The use of a convenient sample and the small sample size are the most prominent threats to the external validity of our study (i.e., the generalizability of the study’s findings) with the approach for accessing the sampling units led it not adequately representative of the entire population. The cross-sectional nature of the study allowed only studying of the correlations between the different variables but not the cause-effect relationships. A similar study relying on a representative larger sample should be conducted using a longitudinal research design.

## Conclusion

The current study revealed that the COVID-19 pandemic has triggered psychological distress among a considerable proportion of the general population in Egypt at an intensity ranging from mild to severe—but, the more psychological distress experienced, the greater the ability to adapt in light of the spread of the SARS-CoV-2 virus. This study also found that the methods of adaptation used by most of the study population relied on emotional-focused coping and the most problem-focused adapted people were the divorced, governmental sector employees, those working in the health care field, and city residents whose monthly income is sufficient to meet their needs. Meanwhile, higher emotional-focused WCS scores were found among private-sector employees, divorced, and younger people.

The primary significance of the assessment of COVID-19-related perceived stress and coping strategy types is paramount at several levels. One of these is the ability to provide appropriate interventions for COVID-19-related perceived stresses and to equip people with suitable strategy types for coping with COVID-19. Besides, it could provide sufficient data for any concerned body, including health professionals, psychologists, policymakers, and planners, in advising the most vulnerable populations on the prevention and control of physiological and psychological impacts of COVID-19, in a timely and appropriate manner. At the onset of major disasters, nationwide strategic planning should be established and delivered through telemedicine for psychological first aid. To reduce psychological distress and limit health problems, a crisis prevention and intervention system should be developed. Furthermore, accessibility to public health services and medical resources should be improved.

## Data Availability

The datasets of the current study are available from the corresponding author upon reasonable request.

## Abbreviations

COVID-19: Coronavirus disease 2019
K10: Kessler Psychological Distress Scale
SARS-CoV-2: Severe acute respiratory syndrome coronavirus 2
WCS: Ways of Coping Scale
WHO: World Health Organization
